# Data on the current-voltage dependents of nickel hollow microspheres based thermo-electrochemical in alkaline electrolyte

**DOI:** 10.1016/j.dib.2020.105770

**Published:** 2020-05-30

**Authors:** Igor Burmistrov, Nikolay Gorshkov, Nikolay Kiselev, Denis Artyukhov, Evgeny Kolesnikov, Bekzod Khaidarov, Andrey Yudni, Gopalu Karunakaran, Eun-Bum Cho, Denis Kuznetsov, Alexander Gorokhovsky

**Affiliations:** a National University of Science and Technology MISiS, Leninskiyprospekt 4, 119049 Moscow, Russian Federation; b Engineering Center, Plekhanov Russian University of Economics, 36 Stremyanny Lane, Moscow, 117997, Russian Federation; c Yuri Gagarin State Technical University of Saratov, Politechnicheskaya street 77, 410054 Saratov, Russian Federation; d Biosensor Research Institute, Department of Fine Chemistry, Seoul National University of Science and Technology, Seoul 01811, Republic of Korea

**Keywords:** Thermo-electrochemical cell, Renewable energy, Seebeck coefficients, Waste heat harvesting, Nickel hollow microspheres

## Abstract

Low-grade waste heat harvesting and conversion into electric energy is an important way of renewable energy development and thermo-electrochemical cells are promising devices to solve this problem. In this paper, we report original data on the current density and maximum output power dependents on voltage of the thermos-cells with nickel hollow microspheres electrodes and different electrolyte concentration (from 0.1 to 3.0 mol/l)which exhibit excellent hypothetical Seebeck coefficient and accordingly high open-circuit voltage values at low source temperature. The composition, microstructure and morphology of the hollow nickel microspheres based electrodes are included here. Because of the low cost of nickel based thermo-cells could be commercially feasible for harvesting low-quality thermal energy, in this connection, the raw data of measurements of their properties are given here.

The data is related to “High Seebeck coefficient thermo-electrochemical cell using nickel hollow microspheres electrodes”, Burmistrov et al., Renewable Energy, 2020 [1].

Specifications Table

**Table:** 

**Subject**	Materialschemistry
**Specific subject area**	New electrode material and construction for thermo-electrochemical convertors
**Type of data**	Figures, Tables
**How data were acquired**	The current-voltage characteristics were measured under potentiodynamic mode
**Data format**	Raw and analyzed data
**Parameters for data collection**	Voltammetry: cold end in room temperature, hot end in isothermostat with temperature up to 75°C, cell discharged with counter voltage growing from 0mV to cell voltage by 5mV/s rate
**Description of data collection**	Electrode fabrication: 0,3 g each pressed with 0.5 t in 12 mm form Electrolyte parameters: potassium hydroxide aqueous solution in various concentrations: 0.1, 0.5, 1, 2, 3 mol/l
**Data source location**	Yuri Gagarin Saratov State Technical University Saratov, Saratov region, Russia
	51°31′50.0"N 45°58′43.0"E
**Data accessibility**	Data are included in this article
**Related research article**	I. Burmistrov, N. Gorshkov, N. Kovyneva, E. Kolesnikov, B. Khaidarov, K. Gopalu, C. Eun-Bum, N. Kiselev, D. Artyukhov, D. Kuznetsov, A. Gorokhovsky, High seebeck coefficient thermo-electrochemical cell using nickel hollow microspheres electrodes, Renewable Energy, https://doi.org/10.1016/j.renene.2020.04.001

## Value of the data

• The data set directed to use by researchers interested in developing new renewable energy sources and understanding the mechanism of metal nickel electrodes working in thermo-electrochemical cell, supercapacitors, batteries, etc.
• The open-circuit potential of the electrolyte concentration for the hollow Ni-microspheres based thermos-cells could give an insight into the chemistry of the electrode process and could help to explain extremely high Seebeck coefficient.
• Comparison with data acquired for other electrochemical couples may be done as well as further experimental studies in the field of increasing the range of electrolyte concentrations.

## 1 Data

**Fig. 1:**
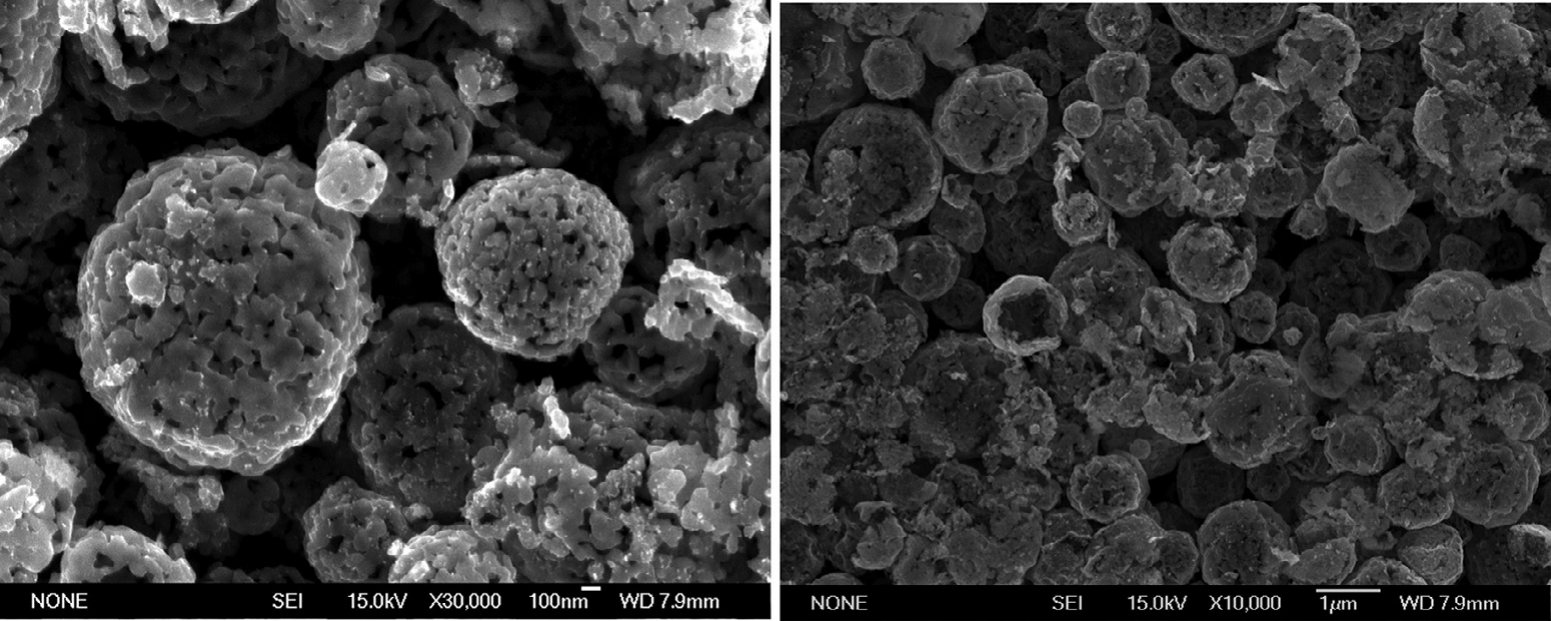
SEM micrograph of nickel hollow microspheres

**Fig. 2:**
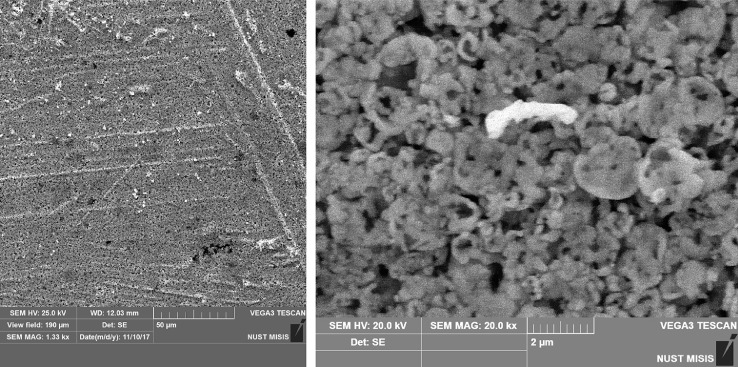
SEM micrograph of electrode surface after pressing

**Fig. 3:**
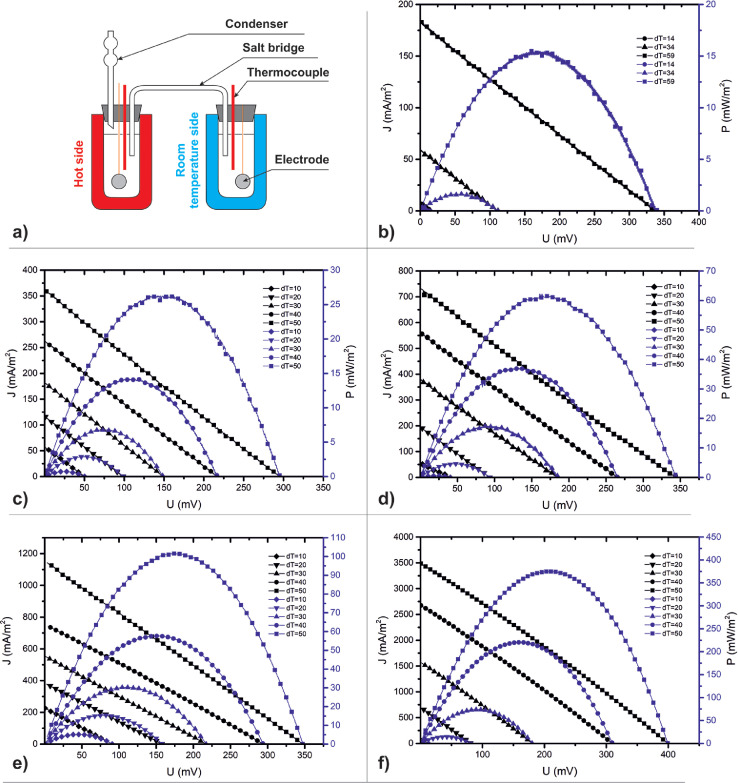
Diagram of a cell with an ion-conducting bridge – a) and load curves of cells with electrolyte concentrations: b) 0.1 mol/l, c) 0.5 mol/l, d) 1 mol/l, e) 2 mol/l, f) 3 mol/l at various temperature differences

The data set in this article describes the electrode materials based on nickel hollow microspheres produced by ultrasonic spray pyrolysis. The hollow structure and particle size of microspheres after synthesis are presented in Fig. 1. Surface morphology of the tablet electrodes are presented in Fig. 2. The measurements were made on the installation shown in Fig. 3a and the load characteristics of these cells with different electrolyte concentrations and at various temperature gradients are presented in Fig. 3. The obtained values of the hypothetical Seebeck coefficient and maximum specific power of the cell for each concentration and temperature difference are presented in Table 1.

**Table 1:** The obtained values of the Seebeck coefficient and maximum specific power

	Concentration, mol/l				
	0,1	0,5	1	2	3
S_e_, mV/K	5.7	5.9	6.8	7	8
P_max_, mW/m^2^	16	26	62	101	374

## 2 Experimental design and methods

The measuring cell is classic for electrochemistry and consists of two flasks with electrodes connected by an ion-conducting bridge (Fig. 3). The hot electrode is thermostated in the LOIP FT-211-25 cryothermostat with an accuracy of 0.1°C. The cold electrode remains at room temperature without active cooling. The used electrolyte in the experiment is aqueous KOH solution with concentrations of 0.1, 0.5, 1, 2, 3 mol/l.

Electrodes weighing 0.3 g are pressed in a mold with a diameter of 12 mm at 0.5 tons. Low pressing force allows to form the electrode without harming the structure of the microspheres. The electrode is placed in a flask on a thin stainless steel wire, which acts as a current lead.

The load curves were obtained with the ELINS Pi-50-Pro potentiostat-galvanostat in the potentiodynamic mode. The parameters of the potentiodynamic mode were established as follows: the counter potential increased from 0 mV to the open circuit voltage at a rate of 5 mV/s.

SEM analysis of nickel microsphere powder (Fig. 1) made it possible to discover the initial structure and the characteristic size of the microspheres. SEM analysis of the electrode surface (Fig. 2) confirmed minor changes in the surface morphology and minor changes in the specific surface area of the tablet shape electrodes after pressing relative to the microsphere powder.

Fig. 3(a) shows a classic diagram of electrochemical cells with an ion-conducting bridge. The use of this circuit is advantageous due to the precise control of the temperature of the electrodes. In case of low electrolyte concentration (0.1 – 3 mol/l) there is no point of inflection on the load curves compared to high electrolyte concentration of more than 6 mol/l [1] and in comparison with nickel sulfate electrolyte [2].

The presented current-voltage characteristics (Fig. 3(b)-(f)) of cells with different electrolyte concentrations showed a correlation between the electrolyte concentration and the maximum specific power and Seebeck coefficient. The values of the Seebeck coefficient and maximum specific power are presented in Table 1.

## Declaration of Competing Interest

The authors declare that they have no known competing financial interests or personal rela- tionships that could have appeared to influence the work reported in this paper.
